# How I manage luspatercept in transfusion‐dependent beta‐thalassemia

**DOI:** 10.1002/hem3.70315

**Published:** 2026-02-19

**Authors:** Daniele Lello Panzieri, Natalia Scaramellini, Simona Leoni, Ali Taher, Maria Domenica Cappellini, Irene Motta

**Affiliations:** ^1^ Università degli Studi di Milano Milan Italy; ^2^ Department of Clinical Sciences and Community Health, Dipartimento di Eccellenza 2023‐2027 University of Milan Milan Italy; ^3^ Fondazione IRCCS Ca' Granda Ospedale Maggiore Policlinico, SC Medicina ad Indirizzo Metabolico Milan Italy; ^4^ Division of Hematology and Oncology, Department of Internal Medicine American University of Beirut Medical Center Beirut Lebanon

## Abstract

Beta‐thalassemia is an inherited anemia characterized by a broad spectrum of clinical manifestations. The most severe form is transfusion‐dependent β‐thalassemia, in which patients need regular blood transfusions to survive, since no adequate amount of hemoglobin is produced by the bone marrow. The therapeutic landscape for this disease is constantly evolving, and new therapies have been recently approved. Luspatercept is the first and only approved drug for treating anemia in transfusion‐dependent β‐thalassemia. Most available information regarding its safety and efficacy is derived from clinical trials, with limited data on real‐world experiences. Thus, a significant gap remains in the literature concerning patient management in everyday clinical settings, particularly in terms of assessing efficacy and the challenges that arise when managing luspatercept. Indeed, effectiveness evaluation in the real‐world presents a much more complex scenario compared to clinical trials. In this paper, we present five clinical cases that drive us through the complexity of luspatercept management and highlight the following topics: (1) the role of genotype in the patient selection, (2) the importance of patient empowerment and the psychological aspects when introducing a new therapy, (3) efficacy assessment in the real‐world, including improvement of iron balance, optimization of pretransfusion hemoglobin, and (4) the importance of the constant monitoring for safety and for adverse events. Emerging evidence and insights from real‐world settings play a crucial role in shaping best practices for everyday clinical practice.

## INTRODUCTION

Luspatercept is a novel erythropoiesis‐modulating agent approved by the European Medicines Agency (EMA) and the Food and Drug Administration (FDA) for the treatment of anemia in transfusion‐dependent β‐thalassemia (TDT), and more recently, by EMA, for nontransfusion‐dependent β‐thalassemia (NTDT).^1^, ^2^ In Italy, a nation with a significant number of β‐thalassemia patients,^3^ at the time of writing, luspatercept is reimbursed by the national health system only for adult TDT; therefore, our real‐world data derive from these patients. In our real‐world cohort, the proportion of subjects who achieved the primary endpoint of the Phase 3 trial was similar to that of the trial,^4^ and comparable results were observed in a recently published national study.^5^ Long‐term data from the BELIEVE trial showed that 77% of subjects achieved one of the secondary endpoints, namely a reduction of the transfusion burden ≥ 33% in any 12‐week interval, with over 70% experiencing at least four response periods.^6^ However, evaluating efficacy in real‐world settings is more complex and should be tailored to each patient according to the individual goals established before treatment initiation. Indeed, in contrast to a randomized clinical trial that requires a standardized and comparable approach, real‐world settings confront significant variability in patients' characteristics and unmet clinical needs. We herein discuss the patient journey through clinical cases from our experience, focusing on the complexity of effectiveness evaluation, which is necessary for appropriate and sustainable treatment implementation.

## SELECTING PATIENTS FOR LUSPATERCEPT TREATMENT

### Case 1

A 48‐year old female TDT with a β^0^/β^0^ (codon39/codon39) genotype, intact spleen, a transfusion burden of 21 packed red blood cell (pRBC) units in 24 weeks (0.88 unit/week; iron intake 0.32 mg Fe/kg/day) and a mean pretransfusion hemoglobin (Hb) of 9.0 g/dL in the 24 weeks before treatment initiation, started luspatercept at the dose of 1.0 mg/kg every 3 weeks and increased at 1.25 mg/kg at the sixth administration. After 48 weeks of treatment, a reduction of at least 33% in transfusion burden was observed in two 12‐week intervals, together with an increase in mean pretransfusion Hb from 9.0 to 9.6 g/dL. Despite the severe genotype and phenotype with significant transfusion requirements, the patient displayed a satisfactory response that lasted over time. Her baseline HbF was 9.2% (equal to 0.89 g/dL).

Patients' selection should be balanced according to local access to treatment and the facility's capability to manage treatment properly. Luspatercept should be offered to all eligible patients, based on the drug product information, local regulations, and the individual risk–benefit ratio, whenever no limitations related to cost or accessibility exist. TDT patients are defined in this paper as patients who received at least 6 units of pRBCs over the 24 weeks before treatment and with no transfusion‐free period of >35 days.

Currently, the only known predictor of response is the baseline pre‐treatment fetal hemoglobin (HbF) level. We demonstrated that TDT patients with a pre‐treatment HbF < 0.6 g/dL are unlikely to respond, with a negative predictive value of 92%,^4^ and these data have recently been confirmed on a larger cohort.^5^ According to the subgroup analysis from the long‐term follow‐up of the BELIEVE trial, patients respond regardless of their age, spleen status, genotype, iron status, and pre‐treatment transfusion burden.^6^, ^7^

As shown in *Case 1*, based on the available data, we do not recommend prioritizing patients according to genotype or transfusion requirements, as the response can vary among individuals. While HbF is a valuable predictor of treatment response, we do not recommend using baseline HbF levels to exclude patients from receiving treatment. Instead, we suggest considering it in cases with a complicated risk–benefit analysis. Since luspatercept treatment carries risks of adverse events (AEs), for example, thromboembolic events (TEEs) and hypertension, it is important to weigh these risks against potential benefits. Evaluating baseline HbF levels can assist in determining whether the treatment is likely to be effective. For patients with an HbF level of less than 0.6 g/dL and a risk–benefit ratio that leans more towards risks than benefits, we advise against starting treatment, especially if alternative options are available. Conversely, even if a patient has a high baseline HbF level (greater than 0.6 g/dL), we cannot overlook the potential risks of AEs associated with this treatment. This is because (1) a high baseline HbF does not diminish the risks of AEs from treatment, and (2) ensuring patient safety must always be the primary concern of clinicians.

## SHARING THE TREATMENT PLAN WITH THE PATIENT

### Case 2

A 21‐year‐old TDT male, genotype β^0^/wt (IVS I‐1/wt) associated with triplication of the α‐globin gene, who phenoconverted from NTDT at the age of 6, began luspatercept at the age of 19 years. His transfusion burden in the 24 weeks before the beginning of the therapy was 12 units (0.5 units/week, iron intake 0.19 mg Fe/kg/day) with a mean pretransfusion Hb of 8.6 g/dL. He rapidly became transfusion‐independent, with a sustained increase of 2 g/dL in Hb level at a luspatercept dose of 1 mg/kg. However, after five doses, the patient decided to interrupt the treatment due to bone and muscle pain and fatigue. Three weeks after the suspension, Hb dropped, and he restarted receiving regular transfusion support (Figure S1). Seven months later, the patient reconsidered his expectations about the treatment and the potential impact of transfusion independence on his lifestyle and quality of life. Also, he explained to the medical team that at the first attempt, his motivation was weak, being more focused on personal and educational issues rather than health care. Therefore, in agreement with his physicians, the patient reconsidered luspatercept treatment and restarted the therapy. The patient is currently transfusion‐independent (treatment duration 88 weeks) and could be considered phenoconverted back to NTDT, with a mean Hb level above 10 g/dL at the dose of 1 mg/kg.

Before initiating therapy with luspatercept, we suggest a multiple‐step approach for each patient:

1. Medical history: thoroughly review the medical history and current status of the patient with a risk–benefit perspective;
2. Patient empowerment: explanation of the evidence from the clinical trial and real‐world experiences, focusing on highlighting the benefits and risks of luspatercept, and discussing patient expectations;
3. Individualized goals and expectations: establish patient‐tailored objective(s) and the potential clinical outcomes and related psychological impact; and
4. Logistic aspects: share the patient's journey, the schedule for luspatercept treatment (every 21 days), and the need for the Hb evaluation before drug administration. At least initially, the transfusion schedule and drug schedule may not overlap; therefore, the patient should be advised that the number of hospital visits may increase.

As shown in *Case 2*, the patient's motivation and empowerment are crucial. In this case, at the first attempt, the patient was not focused enough on the treatment due to different priorities in his life. Most likely, the combination of mild side effects and his scarce motivation led to discontinuation despite the excellent response. After a few months, he realized the potential impact of transfusion independence on his life. After discussing the possibility of restarting treatment with the medical team, evaluating the benefits and risks, and reviewing the hospital appointment schedule, he resumed luspatercept treatment, achieving a successful outcome that positively impacted his quality of life.

Considering that a significant proportion of patients are young adults, another relevant topic to discuss before treatment initiation is family planning. This topic should be addressed before treatment initiation and periodically investigated. Studies on animal models demonstrated the reproductive toxicity of luspatercept.^1^, ^2^ Women of childbearing potential should utilize contraceptive methods during therapy and for at least 3 months after treatment withdrawal before attempting to conceive. In conclusion, as suggested by Origa et al., the loss of a regular transfusion schedule and/or transfusion independence,^5^ together with the impact on life aspirations and objectives, can create anxiety and insecurity. A solid doctor–patient therapeutic alliance is essential to begin therapy under optimal conditions for success.^5^

## EFFICACY ASSESSMENT IN REAL‐WORLD

### Assessment of response based on individual iron balance

#### Case 3

A 35‐year‐old male TDT with a β^+^/β^+^ (IVS1‐110/IVS1‐6) genotype and intact spleen had a transfusion burden of 12 pRBC units in the 24 weeks (0.50 units/week), and a mean pretransfusion Hb of 9.7 g/dL in the 24 weeks before luspatercept initiation. His weight was 50 kg, and his iron intake was 0.24 mg/kg/day. He had a moderate iron overload with a liver iron concentration (LIC) = 3.2 mg/g dry weight (dw) and a mean ferritin of 1024 μg/L. He was started on luspatercept at a dose of 1.0 mg/kg every 3 weeks. The transfusion burden rapidly decreased, meeting the 33% reduction and defining him as a responder. Over the first 48 weeks of treatment, his transfusion burden was 16 units of pRBC (0.33 units/week). The transfusion burden reduction was the one expected by the Phase 3 trial and the label of the drug (−33%), and remained stable during the following weeks. After 1 year, he had a loss of response; therefore, the dose was increased to 1.25 mg/kg with a new sustained response (−38%) in the transfusion burden (Weeks 60–84). The iron intake dropped from 0.24 to 0.15 mg/kg/day.

The primary efficacy endpoint in the BELIEVE trial was the percentage of patients who experienced at least a 33% reduction in transfusion burden during Weeks 13–24, along with a decrease of at least 2 pRBC units over this 12‐week period compared to baseline, which was the 12 weeks before the first dose. Although statistically significant, the impact of such a response was modest (reduction in the luspatercept arm 21.4%), with only one in five subjects qualifying as responders, and similar data were observed in real‐world settings.^4^, ^5^ Per the protocol,^7^ the reason for choosing that specific interval was purely based on statistical considerations, aiming to reduce bias if placebo group subjects dropped out early due to lack of response. More recently, evaluating response over any 12‐week period has become preferred for assessing treatment efficacy.^4^, ^5^, ^6^ We present herein an iron‐centered approach that can be tailored to individual characteristics and objectives.

Regarding the response magnitude, in the Phase 3 trial, a 33% threshold was considered clinically meaningful for regularly transfused patients, based on the expected reduction in iron intake (see supplementary material from the BELIEVE trial).^7^ A direct relationship between LIC and total body iron was demonstrated by Angelucci et al., with a simple arithmetic rule: total body iron (mg/kg) = 10.6 × LIC (mg/g dw).^8^ As shown in some iron chelation studies in β‐thalassemia,^9^, ^10^ a reduction of LIC of approximately 3 mg/g dw over a year is considered a clinically significant change. Therefore, using the Angelucci formula, it is possible to estimate LIC changes following reductions in iron intake. If the therapeutic goal is to achieve a LIC reduction of at least 3 mg/g dw, we can calculate the necessary reduction in iron intake on an individual basis for each patient. This calculation will take into account the transfusion burden and the weight of each patient to determine the corresponding decrease in transfusion burden needed to reach this target.

**Figure 1:**
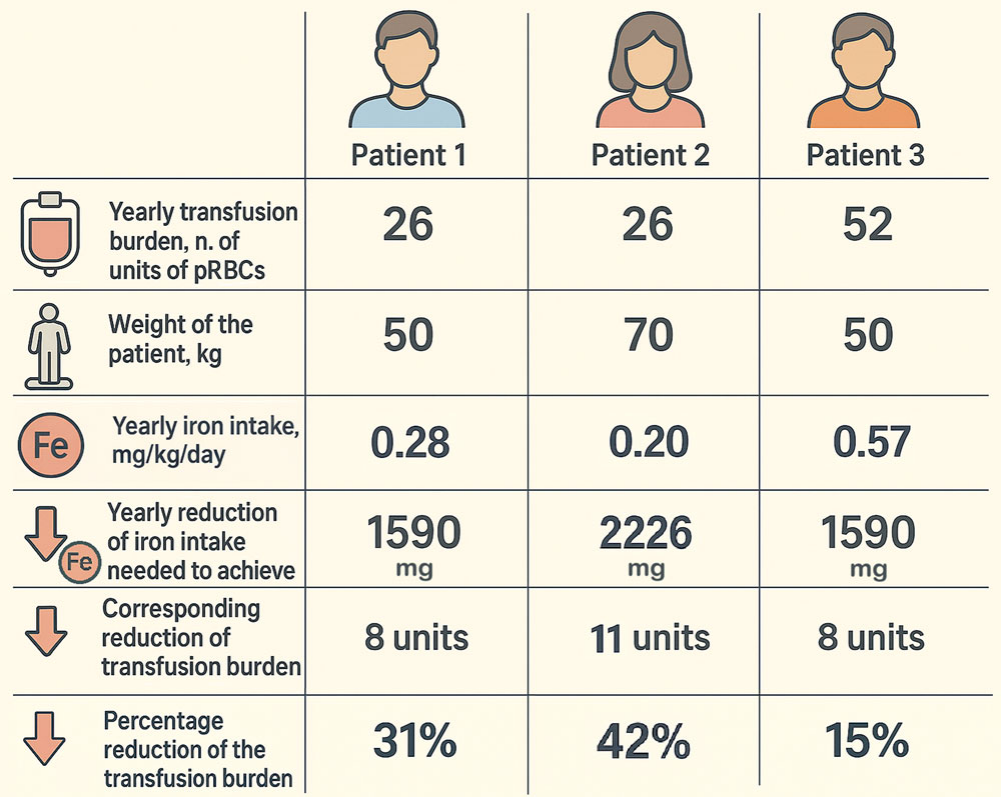
**Individualized transfusion burden reduction based on the Angelucci formula with a target liver iron concentration (LIC) reduction of 3 mg/g dry weight (dw) over 1 year of treatment.** Angelucci formula: total body iron = 10.6 × LIC (mg/g dw). **Patient 1.** Using the Angelucci formula, for this patient, the target reduction of the iron intake over 1 year, necessary to obtain a reduction in LIC = 3 mg/g dw and individualized by weight, is 1590 mg ([10.6 × 3 mg/g dw] × 50 kg). This reduction in the yearly iron intake can be translated into the corresponding decrease in the transfusion burden by dividing it by 200 mg (1590/200 = 8 units). The individualized percentage reduction in the transfusion burden is obtained by dividing the number of units of packed red blood cell (pRBC) needed to be reduced by the number of units of pRBC transfused over 1 year (8/26 = 0.31 = 31%). **Patient 2.** The patient's iron intake reduction over 1 year, individualized by weight, is 2226 mg ([10.6 × 3 mg/g dw] × 70 kg). This yearly reduction of the iron intake translates into an annual reduction in transfusion burden = 11 units of pRBC over 1 year (2226/200 = 11). The corresponding percentage reduction in the transfusion burden over 1 year is 42% (11/26 = 0.42). In this patient, a “classical” response of reduction in the transfusion burden of ≥33% could not meet the expected reduction of LIC. **Patient 3.** The patient's iron intake reduction over 1 year, individualized by weight, is 1590 mg ([10.6 × 3 mg/g dw] × 50 kg). This yearly reduction of the iron intake translates into an annual reduction in transfusion burden = 8 units of pRBC over 1 year (1590/200 = 8). The corresponding percentage reduction in the transfusion burden over 1 year is 15% (8/52 = 0.15).

As in *Case 3*, a 50 kg patient needs approximately 1600 mg of reduction of iron intake (31.8 mg/kg × 50 kg) to achieve a LIC reduction of about 3 mg/g dw over a year, equivalent to 31.8 mg/kg of total body iron. Since each unit of pRBC contains roughly 200 mg of iron, this annual reduction in iron intake would result in a reduction of at least 8 units of pRBC (1600/200 = 8). Thus, in a 50 kg patient who requires 2 RBC units every 4 weeks, or 6 units over a 12‐week period, which is equivalent to 0.5 units per week, reducing 8 pRBC units per year would correspond to a decrease of 2 units every 6 weeks, leading to a 33% reduction in transfusion burden. However, using the same mathematical reasoning, in a 70 kg individual with the same transfusion burden (Figure 1), the same reduction in transfusion burden results in approximately a 2 mg/g dw decrease in LIC over a year, which does not reach the expected clinical significance. Conversely, in a 50 kg individual with a higher transfusion burden (Figure 1), a smaller percentage reduction would be enough to achieve about a 3 mg/g dw decrease. Based on this, for patients whose goal is to reduce iron overload, we can reconsider and calculate the percentage of transfusion burden needed to produce a decrease of 3 mg/g dw (or more, if necessary) over a year. This method allows for response evaluation over a longer and more flexible timeframe. Additionally, this approach focuses more on the patient and is more practical for clinicians. With this new perspective, the treatment response would not be fixed, but tailored to each patient's weight, transfusion burden (and thus iron intake), and expected reduction in the LIC. In summary, depending on individual characteristics, an effective response leading to a LIC reduction of 3 mg/g dw can be achieved with a variable decrease in transfusion burden.

We herein provide a step‐by‐step calculation:

1. Fix a LIC target reduction (at least 3 mg/g dw).
2. Translate it into the equivalent expected reduction of iron intake:
Expectedreductionofironintake=(10.6×LIC×weight(kg))
3. Divide the yearly expected reduction of iron intake by 200 mg (1 unit of pRBC) to obtain the expected reduction of units of pRBC in 1 year:
$$\mathrm{Expected}\;\mathrm{reduction}\;\mathrm{of}\;\mathrm{units}\;\mathrm{of}\;\mathrm{pRBC}=\frac{\mathrm{expected}\;\mathrm{reduction}\;\mathrm{of}\;\mathrm{iron}\;\mathrm{intake}\;(\mathrm{mg})}{200\;\mathrm{mg}}$$
4. Calculate the percentage of reduction in the transfusion burden needed to achieve the target:
$$\mathrm{Reduction}\;\mathrm{of}\;\mathrm{transfusion}\;\mathrm{burden}\;\left(\%\right)=\frac{\mathrm{estimated}\;\mathrm{reduction}\;\mathrm{of}\;\mathrm{unit}\;\mathrm{transfused}\;\mathrm{in}\;52\;\mathrm{weeks}}{\mathrm{units}\;\mathrm{transfused}\;\mathrm{in}\;\mathrm{the}\;52\;\mathrm{weeks}\;\mathrm{before}}$$
5. Evaluate the response based on the individualized transfusion burden reduction.
6. Consider that the patient's response may fluctuate, with phases of reduced transfusion burden exceeding the minimum requirement, and phases in which the reduction falls below the established threshold. Additionally, the relationship between iron intake and iron overload is not always linear. Notably, several factors influence iron metabolism in patients with TDT, including the specific iron chelation drugs used, their dosages, adherence to treatment, and the effects of luspatercept itself.^11^ Other variables, such as transient inflammatory states, can also affect iron metabolism. Therefore, if a response is only nearly achieved within a 12‐week period, it may be advisable to wait and see if a better response occurs during an additional 12‐week period.

### Pretransfusion hemoglobin change

#### Case 4

A 31‐year old male TDT with a β^0^/β^0^ genotype (Cod.8/9 + G/Cod.16‐C associated in cis with mutation Cod.10 C>A), intact spleen, a transfusion burden of 16 pRBC units in the 24 weeks (0.67 unit/week, iron intake 0.32 mg Fe/kg/day) and a mean pretransfusion Hb of 8.2 g/dL before treatment initiation, started luspatercept at the dose of 1.0 mg/kg every 3 weeks and increased the dose to 1.25 mg/kg at the sixth administration due to lack of response. After 36 weeks of treatment (n. 13 injections), no change in transfusion burden was observed (transfusion burden under treatment 0.67 unit/week), while he showed a clinically significant increase in pretransfusion Hb to 9.1 g/dL, despite only a slight increase in the mean transfusion interval from 18.5 to 21 days.

In the BELIEVE trial, changes from baseline in pretransfusion Hb levels were assessed at 12‐week intervals to detect any variations in transfusion practices. Non‐significant changes would have suggested that any reduction in transfusion burden was due to the luspatercept/placebo effect rather than a different transfusion approach. However, in real‐world practice, increasing pretransfusion Hb is a clinically meaningful goal for some patients.^5^ This is specifically true for those with a mean pretransfusion Hb level lower than 9.5 g/dL, which, according to guidelines and recent evidence, is a threshold that indicates a higher risk of mortality in TDT.^12^, ^13^ The higher the pretransfusion Hb, the better the outcome, if iron balance is preserved. To date, the increase in pretransfusion Hb itself is not regarded as an efficacy parameter unless it is linked to a reduction in the transfusion burden of ≥33%.^1^, ^2^ However, in our real‐world experience, for some patients, as in *Case 4*, this could represent a clinically meaningful target to achieve, even if it is not associated with a reduction in transfusion burden.^5^ We believe that increasing pretransfusion Hb levels should be a key efficacy goal.

### Transfusion independence

Table 1 includes the four cases of our real‐world cohort who achieved transfusion independence for at least 8 weeks. All four subjects were initially diagnosed with NTDT and “phenoconverted” to TDT later in life.^14^ All of them have a baseline HbF > 0.6 g/dL. As suggested by Origa et al., the best responders seem to be those with “residual erythropoiesis.”^5^

**Table 1:** Patients who achieved transfusion independence.

	Patient A	Patient B	Patient C	Patient D
Sex	F	F	M	M
Age at diagnosis (years)	6	13	1	4
Age of regular transfusion regimen initiation (years)	6	13	6	28
Genotype	−87/−87	Cod.39/−92C>T	IVSI‐1/wt	Cod.39/IVSI‐6
			ααα/αα	
HbF (%);	32.7;	16.5;	11.4;	34.3;
(g/dL)	3.0	1.4	1.0	3.1
Spleen status	Splenectomy	Splenectomy	Intact	Intact

*Note*: Characteristics of four patients who became transfusion‐independent under treatment with luspatercept. All of them had a mild β genotype and a pre‐treatment HbF > 0.6 g/dL. All of them were not transfusion‐dependent until the age of 6 years (or more), later phenoconverted to TDT. All of them phenoconverted back to NTDT under treatment with luspatercept.

Abbreviations: F, female sex; HbF, fetal hemoglobin; M, male sex.

## TREATMENT INTERRUPTION/DISCONTINUATION

### Case 5

A 48‐year‐old female with TDT phenoconverted from NTDT (β‐globin genotype −87/−87) at the age of 35, had a pre‐treatment transfusion burden of 10 units in the 24 weeks before luspatercept initiation. She had no personal or family history of thrombosis, denied smoking, and had an active lifestyle. She underwent splenectomy at the age of 6 and had primary hypogonadism in substitutive therapy with levonorgestrel/ethinilestradiol. Considering the potential benefits of the treatment in this patient, we decided to start luspatercept. After the first administration, the patient became transfusion‐independent with phenoconversion to NTDT. After nine doses, the patient reported a slight hyperemia of the medial portion of her left thigh without any other signs of local inflammation. Doppler ultrasound (US) of the left leg showed the presence of a partial thrombosis of the left great saphenous vein at 0.5 cm from the saphenofemoral junction with non‐recent aspect. Considering the proximity to the saphenofemoral cross, anticoagulation with rivaroxaban 20 mg per day was initiated. Treatment with luspatercept was interrupted with re‐phenoconversion to TDT. A Doppler US performed after 1 month of treatment showed stability of the thrombus, supporting the hypothesis of a non‐recent event. Considering the risk–benefit ratio of the therapy, in accordance with the patient, we decided to restart luspatercept, obtaining again a phenotypic conversion to NTDT. In agreement with coagulation specialists, we decided to maintain prophylactic low‐dose anticoagulation with rivaroxaban 10 mg per day.

Possible reasons for interrupting or discontinuing treatment include AEs, inefficacy, or logistical difficulties that negatively impact the patient's quality of life. In real‐world experiences, the most common reasons for discontinuation due to AEs were persistent osteoarticular pain, development of arterial hypertension, TEEs (superficial/deep vein thrombosis [DVT] or stroke), and variation in the size or new emergence of extramedullary hematopoietic masses (EMHs).^4^, ^5^

Withdrawing the treatment is mandatory in case of any major AE (e.g., acute liver injury^15^) and pregnancy. A recent report on two unexpected pregnancies during treatment with luspatercept showed normal embryo and fetal growth, body proportions, and organ development.^16^ Regardless of the absence of apparent newborn complications, considering the lack of risk data regarding luspatercept in pregnancy, if the patient is actively trying to conceive or has a positive pregnancy test during treatment, luspatercept should be immediately discontinued. Since it is unclear whether luspatercept metabolites are excreted in human milk, the drug should not be reintroduced until the end of lactation.

A relevant topic with limited data is the occurrence of thrombosis. In the cohort of the Phase 3 trial, TEEs—including DVT, pulmonary embolism (PE), ischemic stroke, and superficial vein thrombosis (SVT)—have been reported in approximately 4% of patients.^6^, ^7^ All patients experiencing TEEs were splenectomized and had at least two additional risk factors for thrombosis development.^1^ Importantly, TEEs were not correlated with Hb levels. Each patient should be carefully evaluated for thrombotic risk beyond the splenectomy, since in real‐world experiences, two thrombotic events occurred in non‐splenectomized patients.^5^ As in *Case 5*, in the case of the occurrence of a TEE, the risk–benefit ratio of continuing the treatment should always be considered in an individualized approach.

Regarding conditions with transient increase in thrombotic risk, in the case of an acute inflammatory or infectious response, although no data are available, we suggest considering a temporary interruption.

Also, although the effect of luspatercept on EMHs is still unclear,^5^, ^6^, ^17^, ^18^ if new EMHs appear during treatment or preexisting ones enlarge, treatment should be evaluated on a case‐by‐case basis. Nonetheless, if any compression symptoms arise due to mass growth, treatment with luspatercept should be promptly interrupted. Careful monitoring of splenomegaly and hypersplenism is essential, as both conditions have been observed in real‐world experience.^5^, ^19^

A common reason for discontinuing luspatercept is inefficacy. Following the drug label, luspatercept should be discontinued after at least five doses (two doses—6 weeks at the starting dose of 1 mg/kg and at least three doses—9 weeks at the maximum dose of 1.25 mg/kg) if a reduction in the transfusion burden is not recorded. We suggest evaluating a prolongation of treatment in agreement with the patient when a subjective improvement in general conditions and fatigue, or if an improvement in pretransfusion Hb is recorded.

## TREATMENT MONITORING

We recommend regular assessment of clinical status, laboratory parameters, and transfusion burden as comprehensive indicators of safety and efficacy. Monitoring should also focus on specific objectives (Table 2). Evaluating changes in transfusion burden can be challenging and time‐consuming in daily clinical routines. Therefore, creating a spreadsheet that includes relevant data on a visit‐by‐visit basis for response evaluation may be helpful. This spreadsheet should cover data from the 24‐week period before starting luspatercept. Using the spreadsheet will facilitate calculations for evaluating the response based on the suggested parameters. A digital spreadsheet could be used according to the country's or hospital's privacy policies.

**Table 2:** Treatment objectives and monitoring strategies.

Objective	How to monitor
Decrease in iron intake	pRBC units over 12 (or 24) weeks Iron intake LIC
Increase in pretransfusion Hb	Pretransfusion Hb
Reduction in the number of transfused units	pRBC units over 12 (or 24) weeks
Reduction in the transfused volume	pRBC unit/transfusion session
Reduction in hospital visits	Transfusion interval
Reduction in the time spent in the hospital per transfusion session	pRBC unit/transfusion session

Abbreviations: Hb, hemoglobin; LIC, liver iron concentration; pRBC, packed red blood cell.

We recommend including at least the following data in the spreadsheet:

– Demographic and clinical data, including age, weight, and blood pressure (to monitor the development of hypertension).
– Luspatercept data: the consecutive administered doses (1, 2, 3, etc.), the dose (0.8, 1.0, or 1.25 mg/kg), and the administration interval.
– Transfusion data: the number of pRBC units transfused, the weight of the transfused units (which could be helpful in calculating iron intake), iron intake, and the interval between transfusions.
– Laboratory parameters, including Hb, red blood cells, reticulocytes and nucleated red blood cells, hematocrit, leukocytes, platelets, creatinine, and liver function tests. Periodical testing of uric acid, folate, and vitamin B12.
– Imaging exams: spleen size, presence, and size of EMHs.
– AEs.

According to EMA indications,^1^ the first reassessment should be scheduled at the third dose to evaluate the need for a dose increase. If a dose increase is necessary, a second reassessment of the response should be done after 9 weeks of treatment at the maximum dose of 1.25 mg/kg. In practice, this tight schedule can be challenging. It is our opinion that the first reassessment after only two consecutive doses of luspatercept is too strict, and in our experience, dose increases are usually performed at the third or fourth dose (9–12 weeks). We recommend calculating the number of units of blood transfused per week (units/week) and comparing it to the number of units/week transfused during the 24 weeks prior to luspatercept treatment.

It is also important to note that luspatercept dosage should be decreased (from 1.25 to 1 mg/kg or from 1 to 0.8 mg/kg) if there is an increase in Hb level >2 g/dL without any transfusion support since the previous dose, or to manage mild AEs.

Safety should be continuously monitored, and in case of AEs, dose reduction, temporary suspension, or interruption should be considered accordingly.

### Iron status parameters

Monitoring iron parameters is essential, and T2* magnetic resonance imaging (MRI) is the most accurate method. According to recent data from the long‐term follow‐up (LTFU) study, a mean reduction of 3 mg/g dw is observed in responders—defined as those who achieve a ≥33% reduction in transfusion burden over any rolling 12‐week period—after 3 years of treatment, rather than after 1 year as expected by the Phase 3 trial protocol.^6^, ^7^ This aligns with evidence of a non‐continuous response. As shown in both clinical trial and real‐world data, most responders alternate periods of reduction above and below 33%, requiring more than a year to achieve the decrease in iron intake needed for a 3 mg/g dw LIC reduction. It is important to note that T2* MRI is not widely available at all sites, and prescription frequency can vary across regions. Therefore, changes in iron intake from baseline are a valuable and cost‐effective way to assess treatment effectiveness. Additionally, luspatercept itself seems to influence iron metabolism, as Garbowski et al. demonstrated that 70% of luspatercept‐treated patients experienced a significant reduction in hepcidin.^11^ Regarding serum ferritin, the LTFU study showed a decline from Week 48 in all luspatercept‐treated subjects, regardless of their response.^6^ Similarly, Origa et al. reported a significant reduction in serum ferritin levels starting early in treatment, with a correlation between ferritin levels and drug response; however, no reduction was observed in LIC measured by T2* MRI.

**Figure 2:**
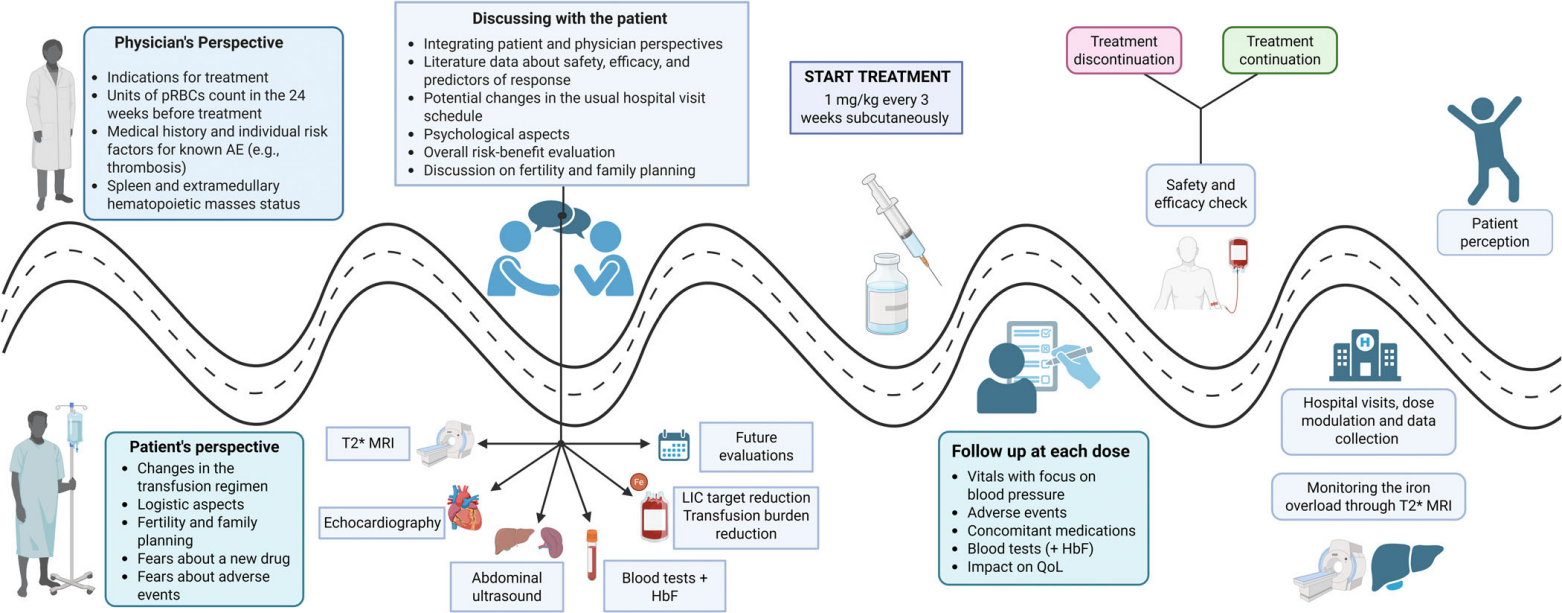
**Patient and physician journey in luspatercept treatment initiation and monitoring.** This figure illustrates the patient and physician journey along an ideal timeline, beginning with the perspectives of both parties before treatment initiation. The process continues with a shared decision‐making phase in which the physician and patient discuss the potential benefits and risks of initiating luspatercept therapy. Once treatment has begun, the journey includes ongoing follow‐up and monitoring, assessing not only safety and efficacy but also the long‐term outcomes associated with luspatercept. AE, adverse event; HbF, fetal hemoglobin; LIC, liver iron concentration; MRI, magnetic resonance imaging; pRBCs, packed red blood cells; QoL, quality of life.

Ultimately, introducing luspatercept calls for a coordinated and patient‐centered strategy that optimizes evaluation, treatment planning, and long‐term follow‐up along the entire patient journey (Figure 2).

## CONCLUSIONS

Managing luspatercept treatment in TDT can pose challenges in the everyday clinical setting. In this paper, we present our approach to managing the treatment with luspatercept from a real‐world perspective, aiming to establish the best therapeutic alliance between physicians and patients. Additionally, we propose a shift in perspective by introducing a new patient‐centered approach to evaluating treatment response. The drug's impact on iron metabolism remains to be clarified through long‐term real‐world experiences.

## AUTHOR CONTRIBUTIONS

**Daniele Lello Panzieri**: Conceptualization; data curation; visualization; writing—original draft; writing—review and editing. **Natalia Scaramellini**: Data curation; writing—original draft; writing—review and editing. **Simona Leoni**: Data curation; writing—original draft; writing—review and editing. **Ali Taher**: Supervision; writing—review and editing. **Maria Domenica Cappellini**: Supervision; writing—review and editing. **Irene Motta**: Conceptualization; supervision; writing—original draft; writing—review and editing.

## CONFLICT OF INTEREST STATEMENT

A.T. reports consultancy fees and research support from Agios Pharmaceuticals, Bristol Myers Squibb, Novo Nordisk, Pharmacosmos, and Vifor Pharma.

M.D.C. reports consultancy fees and research support from Agios Pharmaceuticals, Bristol Myers Squibb, Novo Nordisk, Pharmacosmos, Vifor Pharma, and Sanofi.

I.M. is a scientific advisory board member of Bristol Myers Squibb, Sanofi, and Vertex Pharmaceuticals; received honoraria for lectures from Bristol Myers Squibb, Agios, Vertex Pharmaceuticals, and Sanofi; and research support from Sanofi.

## ETHICS STATEMENT

The study was approved by the ethical review committee of the center “Comitato Etico Milano Area 2” (Protocol Number 317_2022bis) and was carried out in compliance with the principles established in the Helsinki Declaration. Informed consent was obtained from all individual participants included in the study.

## FUNDING

This work was partially supported by the Italian Ministry of Health and Fondazione IRCCS Ca' Granda Ospedale Maggiore Policlinico [RC_2024/25].

This work was partially supported by the funding grant of the Società Italiana di Medicina Interna (SIMI). Open access publishing facilitated by Università degli Studi di Milano, as part of the Wiley ‐ CRUI‐CARE agreement.

## Supporting information


**Supporting Information**.

## Data Availability

The data that support the findings of this study are available on request from the corresponding author. The data are not publicly available due to privacy or ethical restrictions.
