# Supplementary material for: How I manage luspatercept in transfusion‐dependent beta‐thalassemia
Source: Hemasphere. 2026 Feb 19;10(2):e70315. doi: 10.1002/hem3.70315 (PMC12919478; doi:10.1002/hem3.70315)
Supplement: Supplementary file 1 — Supporting Information. [file HEM3-10-e70315-s001.docx]

**SUPPLEMENTARY MATERIAL**

**
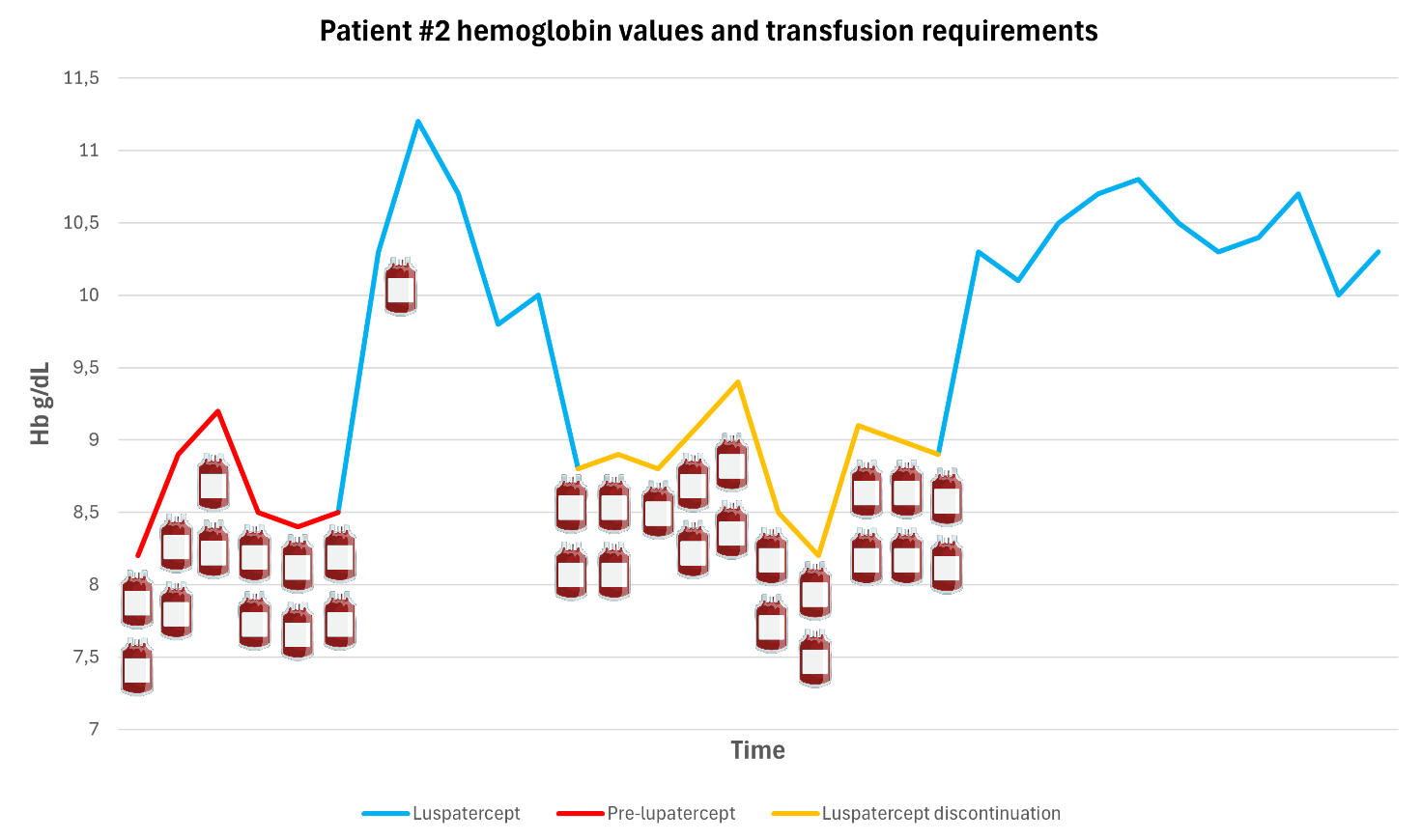
**

**Figure S1**. Graphical illustration of hemoglobin (Hb) levels and transfusion requirement changes in the patient of Case #2. The red line represents Hb levels before initiation of luspatercept, the blue line during luspatercept, and the yellow line during treatment suspension. Before luspatercept, the patient was transfusion-dependent, then, during treatment, became transfusion independent. During treatment, he also maintained a Hb >9.5 g/dL. As treatment was discontinued, Hb levels dropped, and transfusion-dependence recurred. Upon luspatercept re-challenge, Hb levels remained above 10 g/dL, and the patient regained transfusion-independence. Each blood bag icon represents one unit of transfused pRBCs.
